# Protective Effects of Diets Rich in Polyphenols in Cigarette Smoke (CS)-Induced Oxidative Damages and Associated Health Implications

**DOI:** 10.3390/antiox11071217

**Published:** 2022-06-21

**Authors:** Mithun Rudrapal, Siddhartha Maji, Shiv Kumar Prajapati, Payal Kesharwani, Prashanta Kumar Deb, Johra Khan, Randa Mohamed Ismail, Rani S. Kankate, Ranjan Kumar Sahoo, Shubham J. Khairnar, Atul R. Bendale

**Affiliations:** 1 Department of Pharmaceutical Chemistry, Rasiklal M. Dhariwal Institute of Pharmaceutical Education and Research, Pune 411019, Maharashtra, India; 2 RamEesh Institute of Vocational and Technical Education, Greater Noida 201310, Uttar Pradesh, India; rit.siddhartha@rameesh.org (S.M.); rit.shiv@rameesh.org (S.K.P.); rit.payal@rameesh.org (P.K.); 3 Department of Pharmaceutical Chemistry, School of Pharmaceutical Sciences, Shoolini University, Solan 173229, Himachal Pradesh, India; prashantakuamrdeb@shooliniuniversity.com; 4 Department of Medical Laboratory Sciences, College of Applied Medical Sciences (CAMS), Majmaah University, Al Majmaah 11952, Saudi Arabia; j.khan@mu.edu.sa (J.K.); rn.ibrahim@mu.edu.sa (R.M.I.); 5 Health and Basic Sciences Research Center, Majmaah University, Al Majmaah 11952, Saudi Arabia; 6 Department of Microbiology and Immunology, Veterinary Research Institute, National Research Center (NRC), Giza 12622, Egypt; 7 Department of Pharmaceutical Chemistry, MET’s Institute of Pharmacy, Bhujbal Knowledge City, Nashik 422003, Maharashtra, India; ranik_iop@bkc.met.edu; 8 School of Pharmacy and Life Sciences, Centurion University of Technology and Management, Bhubaneswar 752050, Odisha, India; ranjankumar.sahoo@cutm.ac.in; 9 Department of Pharmacology, MET’s Institute of Pharmacy, Bhujbal Knowledge City, Nashik 422003, Maharashtra, India; shubhamk_iop@bkc.met.edu; 10 Sandip Institute of Pharmaceutical Sciences, Nashik 422213, Maharashtra, India; atul.bendale@sandippharmacy.org

**Keywords:** cigarette smoke, oxidative damage, dietary polyphenols, COPD, cardioprotective, bioavailability

## Abstract

Cigarette smoking has been responsible for causing many life-threatening diseases such as pulmonary and cardiovascular diseases as well as lung cancer. One of the prominent health implications of cigarette smoking is the oxidative damage of cellular constituents, including proteins, lipids, and DNA. The oxidative damage is caused by reactive oxygen species (ROS, oxidants) present in the aqueous extract of cigarette smoke (CS). In recent years, there has been considerable interest in the potential health benefits of dietary polyphenols as natural antioxidant molecules. Epidemiological studies strongly suggest that long-term consumption of diets (fruits, vegetables, tea, and coffee) rich in polyphenols offer protective effects against the development of cancer, cardiovascular diseases, diabetes, osteoporosis, and neurodegenerative diseases. For instance, green tea has chemopreventive effects against CI-induced lung cancer. Tea might prevent CS-induced oxidative damages in diseases because tea polyphenols, such as catechin, EGCG, etc., have strong antioxidant properties. Moreover, apple polyphenols, including catechin and quercetin, provide protection against CS-induced acute lung injury such as chronic obstructive pulmonary disease (COPD). In CS-induced health problems, the antioxidant action is often accompanied by the anti-inflammatory effect of polyphenols. In this narrative review, the CS-induced oxidative damages and the associated health implications/pathological conditions (or diseases) and the role of diets rich in polyphenols and/or dietary polyphenolic compounds against various serious/chronic conditions of human health have been delineated.

## 1. Introduction

In 2003, the World Health Organization adopted the Framework Convention on Tobacco Control [1] regarding the effect of cigarette smoking on human health. For decades, the use of tobacco smoking among the humans has remained one of the primary global sources of premature death and disability [2]. The tobacco recrudescence is one of the most serious public health concerns the world has ever faced. According to the WHO statistics, tobacco smoking has been responsible for more than 8 million deaths worldwide per year. In this record, around more than 7 million deaths are the result of direct tobacco consumption through smoking, while 1.2 million deaths have occurred among nonsmokers due to exposure to second-hand smoke [3,4]. Cigarette smoking is a key risk factor for chronic obstructive pulmonary disease (COPD) and a major inducer for the production of oxidants/reactive oxygen species (ROS) in the lungs and in the body of those who are exposed to it [5]. The ground source for this induction is cigarette smoke (CS), which comprises a wide range of chemicals, including a substantial number of oxidizing agents and free radicals [6], that can cause or promote oxidative stress and damage [5,7,8] and which can lead to a number of degenerative pulmonary and cardiovascular diseases, as well as cancer [9,10,11], including the oxidative modification of biological macromolecules such as lipids, proteins, and nucleic acids [9]. It is regarded as a significant factor having an essential role in the etiology of ageing and degenerative disorders [12]. In order to subsist oxidative stress and free radical generation, human bodies have evolved with sophisticated mechanisms for maintaining redox homeostasis. These defense mechanisms fabricated in our body include the scavenging or detoxification of ROS, blocking ROS production, the sequestration of transition metals, as well as enzymatic and nonenzymatic antioxidant defenses, generally known as endogenous [13,14], and others supplied with the diet, namely, exogenous ones. Among the exogenous ones, dietary polyphenols have been examined extensively for their significant antioxidant capacities and other qualities that influence cell functioning [15,16]. Dietary polyphenols belong to a class of secondary metabolites found in a wide range of foods, including fruits, vegetables, wine, tea, extra virgin olive oil, chocolate, and other cocoa-based products. They are predominantly flavones, isoflavones, flavonols, catechins, and phenolic acids derivatives and/or isomers. Dietary polyphenols reveal a multitude range of biologically important functions, including protection against oxidative stress and degenerative ailments. The majority of these biological activities, according to experimental evidence, can be linked to their inherent antioxidant capacities. Dietary polyphenols may provide indirect protection by activating endogenous defense systems and modulating cellular signaling pathways, such as NF-κB activation, AP-1 DNA binding, glutathione biosynthesis, the PI3-kinase/Akt pathway, MAPK protein activation (ERK, JNK, and P38), and Nrf2 translocation into the nucleus [17,18,19]. Epidemiological studies have developed great interest in dietary polyphenols due to their association with health issues and the links affecting them. Large in vitro and in vivo studies have revealed the low bioavailability of polyphenols, along with their beneficial effects [20]. Allicin, present in garlic, whose bioavailability and bioequivalence were found to be unknown, was studied in 13 different subjects. The bioassay was done and the area under 32 h concentration was studied. The bioavailability varied when compared between an empty stomach (36%) and a meal with high protein (22%) [21]. Curcumin showed poor bioavailability, was chemically instable, and had a very poor pharmacokinetic profile. The therapeutic value of it was found to be very poor even if consumed at a high dose (12 g/day). It was found that, because of the low absorption from the intestine and the conjugation metabolism in liver, it gets eliminated through the gall bladder [22]. This review helps to understand the various bioavailability issues related polyphenols and the strategies to overcome them. However, the objective of this narrative review was to address the CS-induced oxidative damages and associated health implications/pathological conditions (or diseases), and the role of diets rich in polyphenols and/or dietary polyphenolic compounds, against various serious/chronic conditions of human health, along with the pharmacokinetic/bioavailability issues of dietary polyphenols.

## 2. Cigarette Smoking, Oxidative Damages and Health Implications

Cigarette smoking currently affects around 10% of the population over the age of 45, but this rises to 50% among heavy smokers, and the cumulative life-time risk of developing COPD is now estimated to be greater than 25%. COPD is increasing most rapidly in low-income countries, where indoor air pollution, such as exposure to biomass smoke, is as common a risk factor as cigarette smoking. Cigarette smoking (CS), which contains 1015 free radicals and 4700 unique chemical components in each puff, is the primary source of inhaled environmentally generated ROS. ROS, such as superoxide anion (O_2_^●−^) and the hydroxyl radical (OH^●^), are very unstable entities that contain unpaired electrons and can cause oxidation and other illnesses [23]. Lungs are continuously getting exposed to oxidants, which are produced as either endogenously created (e.g., by mitochondrial electron transport during respiration or during phagocyte activation) or exogenously produced (e.g., by air pollution or cigarette smoke) [24]. ROS generation has been linked to protein, DNA, and lipid oxidation, which can cause direct lung damage or provoke a variety of cellular responses via the development of secondary metabolic reactive species [25]. ROSs have the ability to induce extracellular matrix remodeling and blood vessel remodeling, boost mucus formation, inactivate antiproteases, cause apoptosis, and regulate cell proliferation [26]. Furthermore, elevated ROS levels have been connected to the activation of transcription factors such as nuclear factor-kappa-B (NF-kB ) [27] and activator protein-1 [28], signal transduction [29,30], chromatin remodeling [31], and proinflammatory mediator gene expression [32]. Cigarette smoking has an uncountable number of effects on human health, though it is only possible to point out a few of them.

### 2.1. Cigarette Smoking and Chronic Obstructive Pulmonary Disease (COPD)

**Figure 1:**
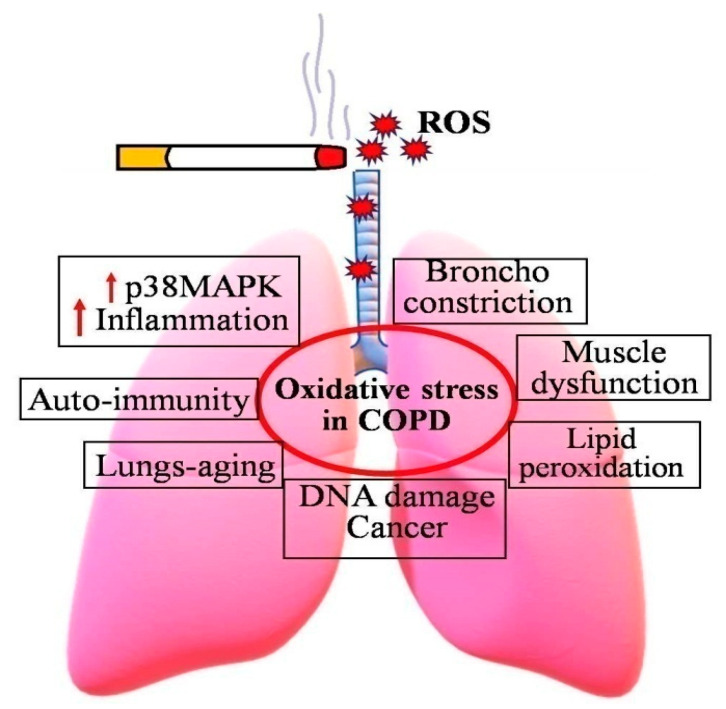
Consequences of increased oxidative stress in COPD patients. Cigarette smoking induces the formation of ROS, and thereby increases incidences of COPD and related diseases.

COPD is a serious global health issue that is currently the third highest cause of mortality and a major source of morbidity. Increased oxidative stress is the main mechanism that drives the pathophysiology of COPD (Figure 1). Some major symptoms of COPD include irreversible blockage of lung airflow, characterized by a gradual fall in forced expiratory volume in one second [33]. Activation of epithelial cells and macrophages, as well as the activation of neutrophils, monocytes, and B and T lymphocytes into the lungs, are all inflammatory hallmarks of COPD [34,35,36].

#### 2.1.1. COPD and Inflammation

In the lungs of COPD patients, around 50 cytokines and chemokines are found [37]. Major intracellular signaling pathways include the transcription factor nuclear factor-kappa-B (NF-kB) and signaling molecules such as Ras/Rac, Jun-N-terminal kinase (JNK), p38 mitogen-activated protein kinase (MAPK), and protein tyrosine phosphatases. Most importantly, NF-kB pathways get activated by oxidative stress, and NF-kB expression and activity get increased in COPD, notably in the airway epithelial cells and macrophages [38]. Oxidative stress also stimulates transforming growth factor (TGF) signaling pathways, which cause small airway fibrosis [39].

#### 2.1.2. COPD and Autoimmunity

Autoimmunity with autoantibodies against epithelial and endothelial cells is becoming more common in COPD lungs, particularly in severe illness [40,41,42,43]. Oxidative stress can produce protein carbonylation (“carbonyl stress”), which results in the formation of neoantigens against which autoantibodies can form. There is evidence of autoantibodies against carbonyl-modified proteins in COPD patients, which may be complement-fixing and hence contribute to lung parenchymal damage [44].

#### 2.1.3. COPD and DNA Damage

Direct DNA damage is caused by oxidative stress. The expression of 8-hydroxy-2-deoxyguanosine (biomarker of DNA oxidative damage) is elevated in the peripheral lungs of COPD patients of cigarette smokers [45]. The failure in COPD repair caused by oxidative stress may explain the higher prevalence of lung cancer in COPD patients compared to smokers without airway obstruction. The failure in COPD repair caused by oxidative stress may explain the higher prevalence of lung cancer in COPD patients compared to smokers without airway obstruction [25,45]. The failure in COPD repair caused by oxidative stress may explain the higher prevalence of lung cancer in COPD patients compared to smokers without airway obstruction [46].

### 2.2. Cigarette Smoking and Lipoprotein Oxidation

Low-density lipoprotein (LDL) oxidation has been found to impart numerous proatherogenic characteristics on lipoproteins in vitro, indicating that the transformation is significant to atherogenesis in vivo [47]. Hypercholesterolemia has been associated to symptoms of increased oxidative stress [48], and statin therapy of hypercholesterolemia has been linked to a reduction in high isoprostanes [49]. In the last couple of years, few research findings have investigated the link between smoking and hypercholesterolemia as separate oxidant stress factors. Endothelium-mediated relaxation is reduced when isolated rabbit arteries are incubated with oxidized LDL from cigarette smoke, but not when treated with natural LDL [50].

Many human investigations have found that LDL obtained from smokers is more prone to oxidation ex vivo than LDL isolated from nonsmokers [51,52]. Furthermore, when LDL is extracted and conditioned 90 min after smoking 6 to 7 cigarettes, it contains more changed LDL than LDL isolated after 24 h of abstinence [53]. Smoking appears to have reversible effects on LDL oxidation [54,55].

### 2.3. Cigarette Smoking and Abnormal Nitric Oxide (NO) Metabolism

The production of NO by endothelial nitric oxide synthase in endothelial cells is essential for the control of proper vascular tone. Even smoking one cigarette has been linked to a reduction in plasma nitrate (an end product of NO) and endogenous vitamin C [56]. These effects were quite temporary and reverted to baseline within 1 h [57,58]. NO bioavailability may be diminished due to decreased generation by a damaged endothelium, as well as increased consumption by the ROS, particularly superoxide. Tobacco use has been linked to enhanced lung neutrophil activation and plasma myeloperoxidase activity [59].

### 2.4. Cigarette Smoking and Thrombogenesis

Cigarette smokers had faster platelet turnover and higher urine thromboxane metabolite excretion, which is a measure of platelet activation in vivo [60,61,62]. Smokers have higher levels of circulating fibrinogen, a risk factor for cardiovascular disease [63,64,65]. Indeed, smoking overrode other factors in predicting fibrinogen levels and attenuated the effects of the fibrinogen genotype [66,67] in a study of genetic polymorphism of fibrinogen genes. Oxidative stress may potentially influence thrombogenesis by nitrative alteration of fibrinogen [68]. Gole et al. [69] discovered that thrombin’s interaction with nitrated fibrinogen nearly doubled, resulting in faster clot formation. Active smoking is related with higher circulating levels of tissue factor [70]. Cigarette smoking may also affect anticoagulant processes [71]. Serum from smokers contains lowers nitric oxide than nonsmokers [72,73].

### 2.5. Cigarette Smoking and Endothelial Dysfunction

Endothelial dysfunction is a precursor to vascular endothelial damage [74]. It is thought to predict unfavorable cardiovascular events as well as long-term outcomes [75,76]. Several investigations have found a link between cigarette smoking and poor endothelial function. On smoking a single cigarette, an acute, rapidly reversible deficit is found [77,78,79].

## 3. Polyphenols: Dietary Sources, Chemistry and Medicinal Importance

Polyphenols are the major group of naturally occurring secondary metabolites that exist in the plant kingdom. They are abundantly available in the various plant parts including fruits, flowers, and leaf of herbs and terrestrial plants. More than 8000 phenolic compounds of diverse structural arrangements have been reported from the plant kingdom [80]. Polyphenols are essentially biosynthesized by plants for the defense mechanism against microbes, environmental stress, and other predators [81]. These phytoconstituents are often found in the plants as a conjugate with one or more sugar moiety and are termed as glycosides. Chemically, they contain one or more phenolic rings with multiple hydroxyl groups on aromatic rings comprising a large number of substitution and structural diversity [82]. Because of the presence of multiple hydroxyl groups, most of these classes of compounds exhibit strong antioxidants and are well known as free radical scavengers. Polyphenols also exhibit wide ranges of biological activities, such as antioxidant, hepatoprotective, antibacterial, anticancer, antidiabetic, antihypertensive, etc., depending on their structural features [83]. Major classes of dietary polyphenols and their sources are depicted in Table 1.

Polyphenols can be broadly categorized into two major groups: flavonoids and nonflavonoids, depending on the number and arrangement of the different phenolic subunits and the linkage of the hydroxyl moiety to the phenolic skeleton [82]. The flavonoid class of compounds can be subdivided into various subclasses based on the degree of oxidation of the heterocyclic ring, including flavones, flavanones, isoflavones, flavonones, flavanols, flavonols, dihydroflavonols, flavandiols, chalcones, dihydrochalcones, aurones, anthocyanidins, proanthocyanidins, biflavonoids, neoflavonoids, etc. [80]. They are richly available in the various plant pigments, whereas the nonflavonoid class is divided into four different subclasses: (1) phenolic acids, (2) stilbenes, (3) lignans, and (4) coumarins [82].

Phenolic acids are the most common nonflavonoid polyphenols, and are further separated into hydroxybenzoic acids (C1-C6 backbone) and hydroxycinnamic acids (C3-C6 backbone) due to the presence of a carboxylic acid group attached to the phenolic ring [83]. They are usually found as a conjugate with a sugar moiety and proteins, and they are hydrolyzable when exposed to acid or alkali. Hydroxycinnamic acid is found in high proportions in a variety of foods and beverages, including wine, tea, coffee, chocolate, vegetables, whole grains, and fruits [84].

Stilbenes have a C6-C2-C6 backbone and are structurally similar to the 1, 2-diphenylethylene nucleus. They can be monomeric or oligomeric. This category includes resveratrol, a naturally occurring essential bioactive molecule [82,85].

Lignans are phenolic compounds with a dibenzyl butane backbone. They are a very rare class of phenolic chemicals. These chemicals are most commonly found in higher plants [82,86].

Flavonoids are the largest group of plant-derived polyphenolic compounds, with approximately 10,000 natural analogues. These are hydroxylated phenolic substances which are synthesized by plants in response to microbial infection. They often exist as bright colored (yellow to red) pigments in the plants and microbes [87,88]. The structural framework of flavonoid compounds comprises a benzo-γ-pyrone ring system (C6-C3-C6 backbone). Structurally, they are characterized as C15 compounds and composed of two phenolic (C6) rings which are linked by a bridge of heterocyclic pyrone rings. Two phenolic rings are denoted as A and B rings, whereas connecting heterocyclic rings is considered as C ring in the structural skeleton [82,90].

The chemical nature and biological potential of flavonoids depend on their structural class, degree of hydroxylation, other functional group substitutions, conjugations, and degree of polymerization, which categorize them in different subclasses (Table 1) [91]. The different category of flavonoids varies in the arrangement of substitution of the C ring, while different compounds within a class differ in the pattern of substitution of the A and B rings. Generally, the B ring is present at the position 2 of the C ring, but it can also be attached in position 3 or 4. Ring B can adopt different structural features, and the three rings can undergo glycosylation and hydroxylation [89]. Flavonoids generally contain three or more -OH groups, which are linked to their structural skeleton and exert to the wide array of the structural configuration. In nature, they are frequently found in glycosylated form with multiple sugar units, termed as flavonoid glycosides [90,92].

The flavonoid ring system (benzo-γ-pyrone ring) contains a double bond between the C-2 and C-3 positions, with a ketone group C-4 of the C ring, denoted as flavones; whereas, in flavanones, the C ring is saturated, containing a double bond between the C-2 and C-3 positions of the ring. These are also called as dihydroflavones [89,91]. Flavonol holds an additional OH group at the C-3 position of the C ring, which makes them more polar than flavone and flavanones. Furthermore, they exist in a dihydroflavonol form and contain a double bond between the C3–C4 positions. Generally, the glycosylation of flavonols occurs in the C-3 position of the C ring [82,91]. Flavan-3-ols, or flavanols containing a hydroxyl group, are always at the C-3 position of the C ring. Unlikely other flavonoids, isoflavonoids contain a B ring attached at the C-3 position of the C ring, whereas, in other flavonoids, the B ring is attached at the C-4 position [82].

Anthocyanidins and anthocyanins are the bright-colored flavonoid compounds. These are positively charged compounds containing flavyliumcations and often occur as chloride salts. Anthocyanidins are the de-glycosylated forms of anthocyanins. The pH acylation and methylation -OH groups connected to the A and B rings, as well as the pH of the environment, influence the color of the anthocyanin compounds [93].

Proanthocyanidins, also known as condensed tannins, are the condensed dimer or trimer of flavanols. During fermentation, they are frequently made from flavanol-rich sources [92].

**Figure 2:**
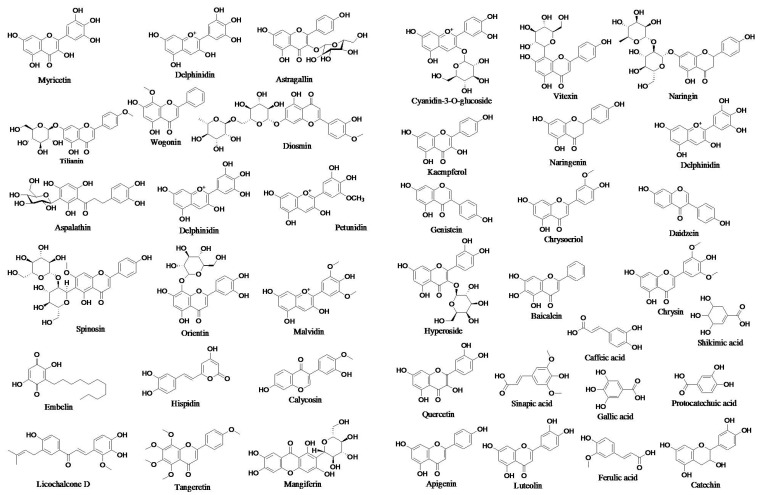
Structures of prototype polyphenols.

Chalcones are open C rings that have flavonoids in them. The chemical scaffold of chalcone molecules is 1,3-diaryl-2-propen-1-one, commonly known as chalconoid [94]. The structures of some prototype polyphenols are presented in Figure 2.

Flavonoids are very much essential to plant for survival during infections, various predator attacks, and environmental stress such as drought. The colors of the different plant parts are dependent on the type of flavonoids present in the tissue [90].

The consumption of flavonoids is proven for various potential therapeutic benefits against human disease [91]. Flavonoid-rich herbal supplements are often found to be significantly effective in the management of hypertension, diabetes, and obesity, along with other complications of metabolic syndrome. In addition, flavonoid-rich food also helps to heal infections, wounds, etc., faster, and improve the immune system [88,91,92]. From the evidence of scientific studies, flavonoids play a remarkable role in the prevention and management of several diseases. A variety of flavonoid molecules have been demonstrated to have many therapeutic benefits, including antioxidant, anticancer, antibacterial, antiviral, antifungal, hepatoprotective, cardioprotective, antidiabetic, analgesic, and anti-inflammatory, etc. [88,95].

Flavonoids exhibit excellent antioxidant properties by scavenging free radicals or chelating with metal ions. These properties are because of the multiple hydroxyl groups (-OH) present in the structure [96]. Flavonoids also activate the Nrf2-HO antioxidant pathway, which provide signals to produce endogenous antioxidant enzymes to maintain the redox balance in the human body during various oxidative stress conditions [97]. Besides the strong antioxidant properties, flavonoids also possess an impressive potential towards the protection of DNA damage and mitochondrial death [98].

Evidently, the approximate intake of 100 mg/day of total flavonoids in the daily diet may reduce the risk of cardiovascular diseases by 6% and 4%, respectively [92]. Recent studies exhibited that polyphenols such as flavonoids improve gut microbial health and maintain healthy gastrointestinal functions [99].

## 4. Protective Effects of Dietary Polyphenols in CS-Induced Diseases

Clinical and epidemiological studies confirm that exposure to cigarette smoke, which is an extremely complex mixture of about 4000 particulate and volatile ingredients, leads to chronic bronchitis, COPD, cancer, cardiac disease, diabetes, eyesight loss, and reproductive problems.

COPD is the most prevalent chronic respiratory disorder, with a remarkable morbidity and death rate. CS includes a variety of toxic components, can trigger alterations in the trachea, lung tissue, and pulmonary blood vessels, and facilitates the onset and progression of COPD [100]. The major processes of COPD development usually involve airway inflammation, oxidative stress, and lung emphysema [101]. A flavonoid “Dihydroquercetin (DHQ)” shows antioxidant and anti-inflammatory properties. DHQ therapy of COPD drastically raised the expression of ferroptosis-related proteins (SLC7A11 and GPx4). The mRNA level of SLC7A11 and GPx4 were likewise upregulated following DHQ therapy and mitigated increased MDA and ROS production, and the markedly reduced superoxide dismutase (SOD) activity as well, which was caused by CSE. In HBE cells, DHQ considerably decreased the enhanced lipid peroxidation caused by CSE. Furthermore, DHQ raised Nrf2 levels in the CS-induced COPD animal model and CSE-treated HBE cells in a concentration-dependent fashion. Interestingly, the enhanced SLC7A11 and GPx4 mRNA and protein levels triggered by DHQ were reversed after HBE cells were administered a Nrf2-specific inhibitor (ML385) [102].

**Figure 3:**
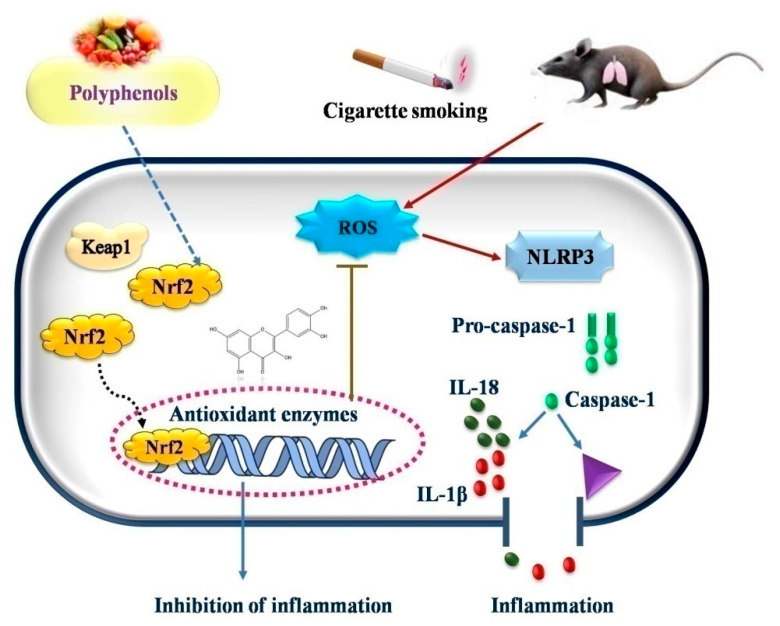
Possible mechanism of action of polyphenols in oxidative stress induced diseases.

Tian et al. (2021) [103] explored the effects of (−)-epicatechin (EC), a flavonoid, on COPD caused by CS. After treatment with cigarette smoke extract (CSE), the EC suppressed the generation of ROS and increased the survival of human bronchial epithelial cells. Western blot was used to determine the expression of superoxide dismutase (SOD), an antioxidant capacity biomarker. In the EC treatment group, the expression of SOD1, SOD2, and SOD3 was considerably higher than in the CSE treatment group. Further research revealed that EC increases the nuclear localization of Nrf2 protein and accelerates ubiquitin-mediated Keap1 degradation by overexpressing tripartite motif-containing protein 25 (TRIM25). Furthermore, EC inhibited NLRP3 inflammasome activation and reduced CSE-induced pyroptosis, as shown by the number of caspase-1-positive cells and by the decreased lactate dehydrogenase production. When Nrf2 was knocked down, the protective effect of EC on human bronchial epithelial cells was partially reversed. EC decreased the activation of the NLRP3 inflammasome and reduced lung inflammation in a COPD rat model, as evidenced by lower interleukin (IL)-1 and IL-18 output (Figure 3).

In the research, it has been observed that EGCG treatment suppressed CSE-induced oxidative stress, as indicated by reduced production and accumulation of ROS in the airway epithelial cells (AECs). Likewise, lipid peroxidation in CSE-stimulated AECs was reduced by EGCG. Moreover, EGCG inhibited nuclear factor-κB activation and the downstream expression of proinflammatory mediators. The findings showed that EGCG had antioxidative and anti-inflammatory effects in CSE-exposed AECs, which is a helpful fact concerning the tea catechin’s potential role for COPD [104].

Flavonoids, such as flavonols (quercetin as glycosides), flavanols ([−]-epicatechin, [+]-catechin), anthocyanins, and specific dihydrochalcones, are found exclusively in apples, as well as many other phenolic chemicals are also present, e.g., chlorogenic acid. The potential benefits of polyphenols in apple were reported by Bao et al. (2013) [105] for the management of COPD. Apple polyphenols at doses of 30, 100, and 300 mg dramatically decreased the development of CS-induced inflammatory cell and gene/protein expression of proinflammatory markers in the lung and bronchoalveolar lavage fluid and considerably alleviated oxidative stress. Treatment with APP also had a significant impact on variance of matrix metalloproteinases-9/tissue inhibitor of metalloproteinase-1 expression which was caused by CS in lungs [105].

In COPD mice, caused by lipopolysaccharide/cigarette smoke (LPS/CS), Zhang et al. (2022) [106] studied the protective effects of naringin against pulmonary endothelial permeability and airway inflammation. Naringin reduced pulmonary histopathological injury and inflammatory cell infiltration as well as cytokine release in bronchoalveolar lavage fluid. Naringin suppressed the intensity of Evans Blue fluorescence in lung tissues while increasing tight junctional protein expression. Interestingly, naringin decreased the number of neutrophils, lymphocytes, and platelets, as well as MDA levels in the blood, while increasing the expression of Aquaporin1 (AQP1) in lung tissues. The percentage of inflammatory cells and mediators’ level were partially recovered after treatment with naringin. The findings showed that naringin decreased inflammatory cell recruitment and MDA content generation in the blood.

Mediterranean diets are well known for their protective effects on inflammation, endothelial dysfunction, and cardiovascular health. Mediterranean diets exert synergistic action with different foods on the vascular endothelium. Extra virgin olive oil (EVOO) is high in flavonoids, phenols, polyphenols, and squalene, and the majority of them have considerable antioxidant activity. Polyphenols contained in olive leaves are anti-inflammatory and protect DNA from free radical damage. Regular EVOO consumption rich in monounsaturated fatty acid (MUFA) and polyunsaturated fatty acids (PUFA) reduces the incidence of macrovascular complications and downregulates the production of inflammatory proteins such as C reactive protein and interleukin-6 [107].

Dos Santos et al. (2013) [108] planned a study to evaluate the effects of curcumin-loaded lipid-core nanocapsules (C-LNC) suspension in CS-induced neurocognitive abnormalities. A poor recognition score increased the oxidative/nitrosative stress biomarkers such as TBARS and NOx and impaired the antioxidant responses, such as the NPSH concentration and SOD activity, were all linked to CS exposure. From the results, it is clear that cognitive deficits, redox imbalance, and variations in ATPase activity were all averted with both free curcumin and C-LNC. Curcumin’s preventive mechanism against smoking cognitive impairment involves maintaining ion homeostasis and redox equilibrium. The data revealed that both free and nanoencapsulated curcumin were efficient in lowering the decreases in Na^+^, K^+^, and Ca2^+^-ATPase caused by CS exposure; however, the dosage of 12.5 mg/kg only provided limited protection. Despite its modest dose (4 mg/kg), the C-LNC showed outcomes that were comparable to the larger doses, which might be due to the enhanced bioavailability (Dos Santos et al., 2013). All these potential advantages from curcumin therapy could be credited to its outstanding antioxidant capabilities, particularly in brain tissue [109,110].

In another study, dos Santos et al. (2011) investigated the activity of the enzymes NTPDase and acetylcholinesterase in lung lymphocytes and peripheral lymphocytes from CS-exposed rats treated with curcumin in another investigation. The rats were given CS and curcumin once a day for five days a week. Curcumin therapy suppressed changes seen in the CS-exposed rats, such as decreased ATP and ADP hydrolysis in lung lymphocytes and peripheral lymphocytes, and increased AChE activity in peripheral lymphocytes. Because it shields membrane lipids from reactive species, its antioxidant function may assist in maintaining normal amounts of extracellular nucleotides. In the test group exposed to CS and treated with 12.5 mg/kg, 25 mg/kg and 50 mg/kg of Cur, histopathological results demonstrated a normal appearance of the tissue, with partial diffuse alveolar septal thickening, incidental peribronchial lymphoid agglomerates, and persistent interstitial infiltrate [111].

The preventive properties of *Juglans regia* (walnut) kernel extract towards CSE- provoked pulmonary toxicities were examined by Qamar and Sultana (2011) [112]. In bronchoalveolar lavage fluid, *J. regia* extract considerably lowered lactate dehydrogenase, total cell count, total protein, and raised glutathione levels. In lung tissue, it also restored glutathione reductase, catalase, and decreased xanthine oxidase activity. *J. regia* kernel extract had a total polyphenolic content of 96 + 0.81 mg gallic acid equivalent (GAE)/g dry weight of extract. The DPPH assay of the extract showed remarkable free radical scavenging potential. When compared to the control group, CSE injection resulted in considerable increases in the total cell count of the rats’ BALF (group I). Doses 1 and 2 (50 and 100 mg/kg b.wt.) of *J. regia* considerably reduced the total cell count levels. When compared to the control group, CSE administration resulted in considerable increases in the total cell count of the rats’ BALF (group I), and doses 1 and 2 (50 and 100 mg/kg b.wt.) of *J. regia* drastically reduced the total cell count.

CS affects biochemical changes in the plasma and blood. For this, Begum et al. (2017) [112] performed an in vitro experiment revealing the free radical scavenging efficacy of green tea polyphenols. The findings revealed altered hematocrit, hemoglobin, plasma glucose, total cholesterol, lipoprotein patterns, and lipid peroxidation, along with vitamins and minerals, and thereafter the activities of a gamma glutamyl transferase, alanine aminotransferase, alkaline phosphatase, and aspartate aminotransferase. Green tea was tested for antioxidants and showed strong free radical scavenging properties. The research findings indicated that green tea supplementation restored the unfavorable changes reported in the biochemical parameters in smokers.

CS has been shown to have significant health consequences on fertility, making it more difficult to conceive. It can also impair the reproductive system and damage the DNA in sperm. Cigarette smoke has already been linked to a variety of diseases, particularly reproductive concerns. *Salvadora Persica* (*S. persica*) is a rich source of polyphenol which was evaluated for defects in reproductive behavior due to CS by Rabbani et al. (2021) [113]. The experimental data demonstrated that CS administration drastically reduced sexual activity indices, while it also boosted blood corticosterone and reduced testosterone levels in rats. The variables associated to libido were considerably improved after the administration of *S. persica* at 200 mg/kg. Changes in the levels of the examined hormones in serum were likewise reversed by the decoction. The activity might be indicative of the presence of several phytoconstituents, including flavonoids, alkaloids, and phytosterols, which may have a vasodilatory effect in the sex organs and augment the production of endogenous testosterone to strengthen libido characteristics that have been debilitated by chronic cigarette smoke exposure.

### 4.1. Vaso-Protective Effect

Zong et al. (2021) [114] investigated the impact of resveratrol on CS-provoked vascular oxidative stress and associated inflammatory disorders. In their study, they reported that endothelial apoptosis contributes to cigarette smoke (CS)-induced diseases such as COPD. Resveratrol shows antiapoptotic activity in CS-induced endothelial cells exposed to destructive stimulus such as the Notch1 (neurogenic locus notch homolog protein 1) signaling mechanism. Further, resveratrol also exhibits a protective effect via regulating autophagy. They also reported that the Notch1 signaling may act as an antiapoptotic factor through regulating the activation of autophagy.

### 4.2. Cardioprotective Effect

Smoking is the leading cause of heart disease, characterized by hyperlipidemia, hypertension, obesity, lack of physical inactivity, and hyperlipidemia. CS increases blood pressure instantly and over time, increases heart rate, lowers blood flow from the heart and the levels of oxygen reaching the body’s tissues, raises the chances of blood clots, weakens blood vessels, and enhances the risk of having a stroke. Atherosclerosis, which is associated to IHD and stroke, is directly linked to hypertension. Oxidative stress is one of the fundamental determinants behind the increased atherogenesis in hypertension individuals. Red wine polyphenols lower blood pressure by promoting nitric oxide synthase (NOS) activity, reducing end-organ damage, such as myocardial fibrosis and aortic thickening [115,116,117].

## 5. Bioavailability Issues of Dietary Polyphenols and Strategies to Overcome

**Figure 4:**
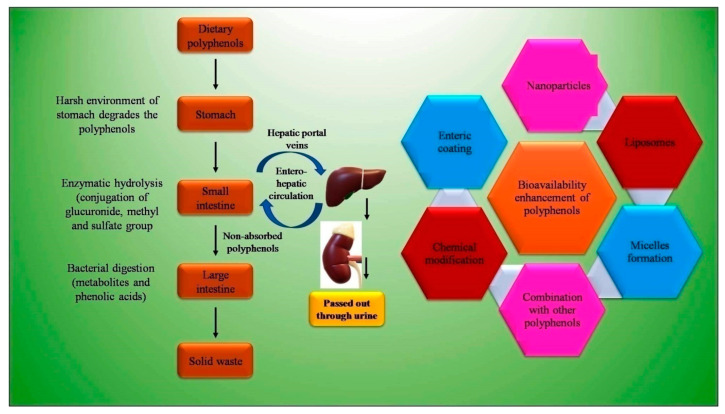
Bioavailability issues of polyphenols and strategies to overcome it.

Bioavailability is an important parameter that determines the presence of a substance (drug or nutraceutical) in the systemic circulation and specific target sites for biological action (Figure 4). Various studies suggest the low bioavailability of dietary polyphenols, with the average oral bioavailability reported on paper at 10%, ranging between 2–20% [118]. The physicochemical properties, such as low solubility, poor GI stability, low intestinal absorption rate, poor intestinal permeability, and permeability across the BBB, of dietary polyphenols mainly contribute to the low bioavailability (Figure 4). Lipinski’s [119] rule explains the physiochemical properties such as lipophilicity having Log P value of less than 5. With the Icase of epigallocatechin-3-gallate (EGCG), present in green tea and having molecular weight of 458 g/ mol and 8 phenolic groups, Lipinski’s rule is not obeyed, and hence the drug is claimed to be poorly absorbed [120]. Low bioavailability is not limited to inefficient physiochemical factors, as other factors, such as rapid metabolism and systemic elimination, enzyme and microbial-mediated biotransformation, and active efflux also results in a low bioavailability. The GIT provides a harsh environment due to the elevated pH, residual dissolved oxygen, metabolic enzymes, and auto-oxidation [121]. Dietary polyphenols are found to interact with salivary proteins, which are rich in proline, through hydrophobic interaction. This results in the formation of soluble polyphenol–protein aggregates, which are precipitated. The precipitation affects the activity of polyphenols, and thus its stability. The high molecular weight of dietary polyphenols also resists its absorption. Tannin, in general, due to this reason, is excreted out of the body in feces [122].

Dietary polyphenols are present in the form of esters, polymers, or in glycosylate. The glycosylation and degree of the polymer is responsible for its low bioavailability. These forms need to be hydrolyzed by the intestinal enzymes or colonic microflora before absorption. The microbiota or enzymes proceed with the deglycosylation, dehydroxylation, and demethylation of the polyphenols and help with the increase in absorption. For instance, dietary flavonoids are poorly absorbed, which undergo deglycosylation by mammalian b-glucosidases for absorption from small intestine [123]. The glycosides are hydrophilic polyphenols with a large size resistant to penetration through the small intestine. The microbial flora in the intestine converts them into the aglycon form, which are highly permeable, as studied in Caco-2 and perfused rat intestinal models, and rapidly absorbed. However, aglycon are poorly soluble (20 µg/mL in water), which is a critical factor for absorption [124].

The encapsulation of dietary polyphenols is desired nowadays to increase the bioavailability for higher bioefficacy. It provides protection against the harsh environment in the stomach and intestine. The reported delivery system for encapsulation is the emulsion-based system, liposomes, nanoemulsion, nanoparticles, protein-based particles, micelles, etc. [121]. Nanoparticles which improve the stability and bioavailability of polyphenols have been reported many papers. Liposomes are found to be a very effective drug delivery system for the delivery of curcumin [125]. The stability of quercetin is also increased in the stimulated gut by the encapsulation in liposomes [126]. Table 2 depicts various bioavailability enhancement techniques in different animal and human models.

## 6. Conclusions

In conclusion, cigarette smoking is considered as a potential risk factor for increased OS and associated tissue damages and diseases. The inflammation triggered by OS is the primary cause of many chronic human diseases. Polyphenols found in our foods/diets have been reported to possess a potential anti-inflammatory effect along with an antioxidant or radical scavenging action. Polyphenols inhibiting OS-associated inflammatory mechanisms/molecular signaling pathways exhibit protective roles against CS-induced inflammation-mediated chronic disorders such as COPD and other lung diseases. In view of having such protective functions, diets rich in polyphenols/dietary polyphenols can be developed as therapeutic molecules with promising antioxidant and anti-inflammatory properties. In this context, further understanding of the molecular mechanisms presumably involved in the protective roles of polyphenols in various health problems is required to be explored.

## Figures and Tables

**Table 1:** Classification of polyphenolic compounds and their main dietary sources with examples [84,85,86,87,88,89].

Class of Polyphenols	Subclass	Prototype Compounds	Major Dietary Sources
Flavonoids	Flavone	Baicalein, apigenin, luteolin, chrysin	Herbal tea, fenugreek, onion, garlic, black pepper, citrus fruits, green leafy vegetables
	Isoflavone	Genistein, daidzein, biochanin A, glycitein	Chickpea, peanut, dairy products, eggs, meat, seafood, soy products, legumes
	Flavonol	Rutin, quercetin, myricetin, fisetin	Tea, chocolate, cocoa, onions, scallions, kale, broccoli, apples, berries,
	Flavanonol	Taxifolin, aromadendrin, engeletin	Milk thistle seeds, citrus fruits
	Flavanol	(−)- epicatechin, (+)-catechin, (−)-epigallocatechin gallate (EGCG), theaflavins	Cocoa, chocolate, tea, grapes, apples
	Chalcone	Butein, xanthoangelol, 4-hydroxyderricin, cardamomin, isoliquiritigenin, isosalipurposide	Tomatoes, liquorice, shallots, bean sprouts
	Flavanone	Hesperidin, hesperetin, naringin, naringenin, eriodictyol	Pomegranate, citrus fruits, tomatoes, grape fruit
	Dihydrochalcones	Phlorizin, aspalathin, nothofagin	Apples and apple products, rooibos tea
	Anthocyanidins	Cyanidin, peonidin, delphinidin, petunidin, pelargonidin, malvidin	Red wines, cherries, red grapes, berries, flowers, oranges, black soybeans, hibiscus sp., purple/black rice, onions, red potatoes, purple cabbage
	Proanthocyanidins	Procyanidin B1, procyanidin B2, procyanidin B3	Berries, cherries, red grapes, red wines, flowers, oranges, black soybeans, banana, cocoa, and apricot, cereals such as sorghum and barley
Non-flavonoids	Phenolic acids	Caffeic acid, sinapic acid, gallic acid, protocatechuic acid, ferulic acid, *p*-coumaric acid	Green tea, citrus fruits, kiwi, coffee, berries, apples, rice bran, passion fruit, cherries, mangoes, wheat, corn flours
	Stilbenes	Resveratrol	grapes (skin), mulberries, peanuts, red wine
	Lignans	Silymarin, sylibin, sesamin, syringaresinol, ecoisolariciresinol, matairesinol, medioresinol, pinoresinol, lariciresino	Flaxseed, soybeans, broccoli, cabbage, milk thistle, apricots, strawberries, etc.
	Coumarins	Dicumarol, osthole	Cinnamon, green tea, carrot, bison grass

**Table 2:** Bioavailability issues of polyphenols and various pharmaceutical formulations/delivery systems to overcome it.

Polyphenol	Bioavailability Issue	Delivery System	Subject	Result	Reference
Curcumin	Low bioavailability and degradation in solution form	Microencapsulation of curcumin in liposomes by the combination of ethanol injection and high-pressure processing	-	Effectively decreases the size of particle and PDI, which helps to cross the biological membrane. Sterilizes the bacterial, which prevent degradation in solution	[127]
	Low bioavailability and rapid metabolism	Nanoparticle fabricated by EGCG and PVP	-	Bioavailability increased 12-fold through intestine EGCG Inhibit the metabolism of Cur, Shows high Caco-2 monolayer permeation and cellular uptake	[128]
	Low bioavailability and rapid metabolism	Emulsion was formed using different types of oils: corn oil, olive oil, and medium chain triglycerides (MCT)	-	Type of oil increased its transenterocyte absorption and reduced cellular metabolism	[129]
	Less physicochemical properties and oral bioavailability	Microencapsulating turmeric oleoresin with bioenhancers by spray drying using piperine and quercetin	-	Spray-dried powder with piperine (PIP) and quercetin (Quer) has higher permeability	[130]
	Low solubility and bioavailability	Zein-based nanoparticles	Wistar rats	Incresaed (9-fold) oral bioavailability with respect to the standard curcumin natural extract.	[131]
	Low bioavailability	Curcugen: dispersible, 98.5% turmeric-based curcuminoids formula	Randomized double-blind, 2-way cross over, single oral dose in humans	Auc-39 times and Cmax 16.1 times higher than of curcumin	[132]
	Low bioavailability	Curcumin-encapsulated chitosan (Cur-CS) nanoparticles	Crandell–Rees feline kidney of cat	Enhanced bioavailability, Cmax- 621.5 ng/mL three times more than normal curcumin	[133]
	Low bioavailability	Curcumin-loaded self-microemulsifying lipid carriers	Male Wistar rats	Higher bioavailability (29-fold) as compared to curcumin suspension	[134]
Quercetin	Low bioavailability and less efficacy	Quercetin nano emulsion	Streptozocin-induced antidiabetic study in rats	Cmax of quercetin NE is 5962.74 ± 238.54 ng/mL and of quercetin pure drug is 1634.28 ± 70.18 ng/mL. AUC0-t and AUC0−∞ were 4.46 and 5.32 times higher than pure drug, respectively	[135]
Green tea (Epigallocatechin-3-gallate and L-theanine)	EGCG bioavailability is <5%	Preparation of EGCG + LTA/β-cyclodextrin (βCD) inclusion complexes by freeze-drying EGCG + LTA	Rats	EGCG bioavailability is improved through lipid lowerig and weight loss effects of EGCG (*p* < 0.05)	[136]
	Low permeation and poor stability leads to low oral bioavailability	Nanospanlastic	Male Wistar rats	Cmax- niosomal formula (*p* < 0.05) and free EGCG dispersion (*p* < 0.001). AUC- niosomal formula (*p* < 0.01) and EGCG dispersion (*p* < 0.001)	[137]
	Low bioavailability and chemical instability	EGCG loaded solid lipid nanoparticles SLN	Male Wistar albino rats	Cmax of EGCG is 60.7 ± 1.07 * and EGCG loaded SLN 240 ± 16 * AUC of EGCG is 567 ± 14.5 * while EGCG loaded SL is 2329 ± 434.5 **	[138]
	Poor oral bioavailability	Nanoparticles (NP)	Sprague Dawley rats	Cmax- EGCG NP 653.5 ± 181.3 * and EGCG powder 564.5 ± 121.7 * AUC ^0–∞-^ EGCG NP5,241.6 ± 387.9 ** and EGCG powder 1321.6 ± 201.4 **	[139]
	Poor bioaccesibility	Nanoemulsion	Sprague-Dawley (SD) rats	Cmax- nanoemulsion 166.7 ± 22.6 * and sol 258.8 ± 135.1 * AUC0-t- nanoemulsion 17.1 ± 0.1 ** and sol 13.3 ± 0.2 **	[140]
Green tea (Catechin)	Poor oral bioavailability	Catechin-loaded chitosan-tethered liposomes (Chitosomes)	Male Wistar rats	Cmax- Chitosomes 239.0 ± 35.27 * and sol 120.0 ± 3.97 * AUC^0–24^- Chitosomes 12,183 ± 1760.00 ** and sol 5739 ± 205.50 **	[141]
Flaxseed	Poor efficacy	Flaxseed oil-based neuronanoemulsions (NNEs)	Balb/c mice	Plasma Cmax- NNE 24.09 ug/mL8 and pure drug suspension (PDS) 12.98 ug/ml * AUC0-12- NNE 96.38 ± 1.39 ** and PDS 18.10 ± 0.15 ** Brain Cmax- NNE 12.98 ± 0.05 * and PDS 1.67 ± 0.02 AUC0-12- NNE 107.58 ± 3.75 ** and PDS 13.18 ± 0.25 **	[142]
Gallic, quercetin, amla, pomegranate	Poor bioavailability	Polyherbal nanoparticles and polyherbal extract following oral administration, pharmacokinetic parameters for polyherbal nanop	Male Wistar rats	GA and quercetin in polymeric nanoparticles improve their oral bioavailability	[143]

* Cmax—maximum plasma concentration (ng/mL), ** AUC—area under the curve (ng·h/mL). EGCG—epigallocatechin-3-gallate, PVP- poly (N-vinylpyrrolidone).
